# Emotion-based learning: insights from the Iowa Gambling Task

**DOI:** 10.3389/fpsyg.2014.00162

**Published:** 2014-03-21

**Authors:** Oliver H. Turnbull, Caroline H. Bowman, Shanti Shanker, Julie L. Davies

**Affiliations:** School of Psychology, Bangor UniversityBangor, UK

**Keywords:** emotion-based learning, intuition, prejudice, psychotherapy, episodic-memory

## Abstract

Interest in the cognitive and/or emotional basis of complex decision-making, and the related phenomenon of emotion-based learning, has been heavily influenced by the Iowa Gambling Task. A number of psychological variables have been investigated as potentially important in understanding emotion-based learning. This paper reviews the extent to which humans are explicitly aware of how we make such decisions; the biasing influence of pre-existing emotional labels; and the extent to which emotion-based systems are anatomically and functionally independent of episodic memory. Review of literature suggests that (i) an aspect of conscious awareness *does* appear to be readily achieved during the IGT, but as a relatively unfocused emotion-based “gut-feeling,” akin to intuition; (ii) Several studies have manipulated the affective pre-loading of IGT tasks, and make it clear that such labeling has a substantial influence on performance, an experimental manipulation similar to the phenomenon of prejudice. (iii) Finally, it appears that complex emotion-based learning can remain *intact* despite profound amnesia, at least in some neurological patients, a finding with a range of potentially important clinical implications: in the management of dementia; in explaining infantile amnesia; and in understanding of the possible mechanisms of psychotherapy.

## INTRODUCTION

Over the last few decades, there has been a growing interest in the cognitive and/or emotional basis of complex decision-making (e.g., Bechara et al., 1994; Damasio et al., 1996; Rogers et al., 1999; Manes et al., 2002; Turnbull et al., 2003; Bowman et al., 2005; Peatfield et al., 2012). This interest was, in large part, inspired by the well-established finding that neurological patients with lesions to ventromesial (VM) frontal lobes often showed normal intelligence, with near or near-to-normal performance on a range of “executive” tasks (e.g., Bechara et al., 2000b). However, in spite of these domains of preservation, such individuals often displayed difficulties in learning from past mistakes, with real life manifestations such as entering repeatedly into inappropriate relationships, and unsuitable business agreements. Such decisions may immediately seem rewarding, but typically prove to be counter-productive in the long run, often leading to career termination and financial losses (Damasio et al., 1991; Bechara et al., 2000a). Notably, such individuals display failures in using emotional feedback from previous situations (i.e., the punishing consequences of impulsive actions) in the guidance of their future choices.

Measuring these decision-making failures in the real-world is challenging, both ethically and methodologically. The Iowa Gambling Task (IGT) was developed as a simple neuropsychological tool to tap into such deficits in emotional-processing, which might be associated with complex decision-making difficulties, as observed in individuals with frontal lobe lesions (Rolls et al., 1994; Damasio et al., 1996; Lezak et al., 2012). In a poetic turn of phrase, patients with VM lesions were argued to have “myopia for the future” (p. 217), where their focus was on the *immediate* outcome of decisions, with an apparent indifference to the long-term consequences of their actions (Bechara et al., 1994; Bechara, 2005).

A key element of the recent complex decision-making literature has been the role of *emotion* (Bechara et al., 1994; Damasio et al., 1996; Rogers et al., 1999; Manes et al., 2002), and indeed its ability to drive emotion-based learning (EBL) during complex decision-making (Damasio et al., 1996; LeDoux, 1996, 2000; Turnbull et al., 2003, 2006). EBL systems are known to facilitate insights about the possible outcome of complex decisions, based on prior experience of the emotional consequences of actions, with particular objects and/or agents (Claparède, 1951; Johnson et al., 1985; Tranel and Damasio, 1993; Bechara et al., 1994; Damasio et al., 1996; Rogers et al., 1999; LeDoux, 2000). The role of emotion in such decision-making is supported by studies of patients with VM frontal, amygdala, and insular lesions (e.g., Bechara et al., 1997, 1999, 2003; Clark et al., 2008), as well as studies measuring skin-conductance changes (e.g., Bechara et al., 1996, 1997, 1999; see also Suzuki et al., 2003). Importantly, (see below) this class of memory (or learning) appears to be independent of the episodic memory systems of the medial temporal lobe (Claparède, 1951; Tulving and Schacter, 1990; Turnbull and Evans, 2006; Evans-Roberts and Turnbull, 2011).

## THE IOWA GAMBLING TASK

The IGT (Bechara et al., 1994) has become the key experimental paradigm in evaluation of emotion-based decision-making, especially when humans are faced with emotion-mediated information, ambiguous contingencies, and uncertain consequences (e.g., Rogers et al., 1999; Manes et al., 2002; Bowman and Turnbull, 2004; Happaney et al., 2004). The IGT has been extraordinarily influential, with Bechara et al.’s (1994) original paper having already acquired over 3000 citations on a Google Scholar search for this paper (November 2013). The spread of influence is also remarkably diverse, spanning a range of theoretical, and clinical papers in psychiatry (e.g., Cavedini et al., 2002; Evans et al., 2005; Must et al., 2006), psychology (e.g., Schmitt et al., 1999; Blair et al., 2001), neuropsychology (Turnbull and Evans, 2006; Torralva et al., 2007), and neurology (e.g., Bechara et al., 1999; North and O’Carroll, 2001; Cavedini et al., 2002; Anderson et al., 2006).

A number of psychological variables have been investigated as potentially important in understanding the nature of these EBL systems. The most prominent of these are (i) the extent to which we are explicitly aware of the basis of such decisions; (ii) the biasing influence of pre-existing emotional labels in complex decision making; and (iii) the extent to which EBL systems are anatomically and functionally independent of episodic memory systems. Each of these issues are briefly reviewed in this article.

### DECISION-MAKING OUTSIDE AWARENESS

An important element in our understanding of the nature of emotion-based-learning, and the factors that drive learning on the IGT in particular, is the question of conscious awareness. The Iowa group have argued that the IGT is extremely complex in nature (Damasio, 1994, pp. 205–222), and that participants do not appear to explicitly understand the contingencies of the game (Bechara et al., 1994, 1996, 1997, 2000b). In analyzing this issue, it is important to keep in mind the definition of “awareness” used by the original Iowa group (e.g., Bechara et al., 1997) – an issue which may explain some of the emergent controversies amongst IGT researchers.

Bechara et al. (1997) explored how participants “conceptualized” the task, by which they appear to have meant the broad understanding of the contingency values on the IGT, and the types of (explicit) strategies used on the task. In their study, they asked participants (patients with VM lesions and neurologically normal controls) two questions: “(i) Tell me all know about what is going on in this game? (ii) Tell me how you feel about the game” (Bechara et al., 1997, p. 1293). In other words, they sought a definition of “awareness” which emphasized formal but also general (and, arguably, rational or cognitive) understanding, as well as broadly based feelings about the task. An initial phase of task awareness was labeled as the “*hunch” *period, where neurotypical participants experienced conscious, but poorly formed impressions about the task (Bechara et al., 1997). During this period, neurotypical participants reported “liking” or “disliking” certain decks, often guessing the general contingencies of the decks. During a later phase of the task, most neurotypicals reached a “conceptual” period – developing a better awareness of the rewarding nature of the decks. Notably, after encountering losses on specific decks, (neurotypical) participants developed pre-decision anticipatory skin conductance responses (SCRs). While, neurological patients did not generate these anticipatory SCRs, nor did they tend to enter the “*hunch” *period. In the later periods most neurological patients were unable to shift their pattern of choice away from the “bad” decks though many did develop a conceptual awareness. However, Bechara et al. (2000a, p. 301) reported some instances of the famous “knowing versus doing” dissociation, (first noted by Teuber, 1964) where “… patients “say” the right thing but “do” the wrong thing”. Even more paradoxically, they reported that some neurotypical participants did *not* reach the conceptual period in that they did not describe an awareness regarding which decks were good and which were bad, *yet* they still made increasingly advantageous choices over time (Bechara et al., 1996, 1997).

In sum, they appear to suggest that conscious awareness on the task, and good performance are unrelated. The Iowa group explained the “unconscious” (unaware) nature of these decisions in terms of the somatic marker hypothesis (SMH; ; Damasio, 1989a,b, 1994; Damasio et al., 1991, pp. 205–222; Bechara et al., 2000a), where “bodily” (i.e., extra-cerebral) systems play a role in facilitating decisions (Bechara et al., 1997). This proposition has received some experimental support (Bechara et al., 2000a), but it has also attracted criticism (Tomb et al., 2002; see Dunn et al., 2006 for a review).

Further support for advantageous decision-making occurring *outside *of explicit**awareness, might be argued to come from the “BLINK” task (Peatfield et al., 2012). An analog of the classic IGT, BLINK is some 25 times faster to complete than the conventional computerized IGT (Bechara et al., 1999). Here, individual decisions are presumably so rapid that little opportunity arises for conscious awareness to develop, thus meeting the criteria for “fast and frugal” decision-making (Gigerenzer, 2004). Notably, in spite of the rapid response rate on the BLINK paradigm, participants show IGT-like performance (see Peatfield et al., 2012 for a detailed discussion of BLINK).

In recent years, Bechara’s claim of advantageous decision-making outside of awareness has been shaped by a series of papers which suggest that *some* forms of conscious awareness *are *available to participants on the IGT. The first of these more formal investigations was reported in Maia and McClelland’s (2004) study, based on a structured questionnaire that assessed participants’ knowledge of the IGT. Maia and McClelland probed the general awareness of task contingencies, without asking participants to specify the cognitive details underlying their understanding. Importantly, most participants who made advantageous choices, and thus showed preference for one or more of the decks, also demonstrated conscious feelings about the decks. Indeed, by the end of Block 1 (i.e., 20 card selections made) participants were able to report basic affective properties of decks, and by 50 card selections, the majority of participants could correctly report “good” decks. Such understanding would readily correspond to participants’ decision preference (see Maia and McClelland, 2004).

Their results suggested that when behaving advantageously, participants not only had access to some explicit knowledge about the “goodness” or “badness” of the deck, but also had reportable knowledge that was well placed to facilitate choice (Maia and McClelland, 2004, p. 16078). Maia and McClelland (2004) therefore claimed that participants playing the IGT *did* have access to explicit awareness about the contingencies of the game. They argued that this resulted from the self-paced nature of the task, which allowed ample time for deliberative reasoning, and also that the outcomes of choices were presented in a clear numerical form, which aided explicit tracking and learning of the incentive nature of each deck (at least to *some* degree – though see Peatfield et al., 2012 above). Thus, Maia and McClelland (2004) posited a degree conscious awareness of the task in participants, albeit of a different form of awareness to that proposed by Bechara et al. (1997). Indeed, this difference was captured by asking participants probing questions about the task, rather than by assessing notoriously difficult-to-verbalize and general feeling. Therefore, Maia and McClelland’s (2004) quantitative method successfully examined explicit awareness, but failed to tap affectively mediated *qualitative* knowledge (feelings) about the game that may indeed facilitate favorable choices. Importantly, Maia and McClelland (2004) suggests multiple source of information might possibly guide the choice during complex-decisions.

Further, empirical support on the question of awareness, comes from the work of Bowman et al. (2005). In this study, participants quantitatively rated the “goodness/badness” of each deck after each twenty-card block. Bowman et al.’s (2005) data suggested that participants could explicitly report affective evaluation (i.e., the relative goodness/badness) of the task objects, even during the “pre-hunch” phase (Bechara et al., 1997). In fact, participants showed obvious awareness of the “valence” of the decks, even following the *first* 20 trials of the task. Other studies (e.g., Evans et al., 2004; Cella et al., 2007) using the same method of tracking task subjective awareness, confirm these original findings, and indeed extend them to a psychiatric population (Evans et al., 2005). However, Turnbull et al. (2007) confirmed, in neurotypicals, that dissociations do occur between explicit deck ratings and behavioral choices on the IGT – suggesting that participants can and do actively ignore explicit knowledge regarding the incentive values of their choices, in favor of implicit emotion-mediated knowledge, especially in situations where varying sources of information come into conflict.

Thus, it appears that explicit (emotion-mediated)-knowledge of incentive values of choice is available *much *earlier than originally claimed by Bechara et al. (1997). This form of awareness is also a type substantially different in quality to that encountered during explicit cognitive approaches to decision making (Gilhooly and Murphy, 2005). The descriptions of these decisions emphasize the fact that the non-cognitive choices are, in contrast, poorly formed (“a hunch”) and laden with affect (“a gut feeling”). It is perhaps this knowledge that subserves the phenomenon that has been long described as “*intuition” *(see also Kahneman and Tversky, 1973; Stanovich and West, 2000; Kahneman, 2003; Turnbull et al., 2003, 2005).

## INTUITION?

We are therefore faced with an interesting, and under-investigated, phenomenon, whereby humans are aided in navigating complex and uncertain problem-spaces, via the awareness of emotion-based signals – presumably derived from prior experience of objects and/or agents. (Kahneman et al., 1982; see Kahneman, 2003) have long described the properties of such *intuitive* responses as being fast, rapid, explicit, effortless, and emotionally laden. Stanovich and West (2000) have proposed a similar dichotomy (e.g., Hogarth, 2001; Myers, 2002). Both seek to discriminate between systems underpinning “intuition” versus “reasoning” (Kahneman, 2003). One approach (intuition; or System 1) generates an overall and apparently imprecise general impression of objects or situations, through an involuntary process sometimes described as *natural assessment *(Tversky and Kahneman, 1983). This phenomenon emerges without intention or effort, and could not (they argued) be verbalized explicitly. In contrast, the reasoning pathway (System 2) is involved when more formal *judgements *are made, even if these are not overtly expressed (Kahneman, 2003; for more on this in relation to the IGT see Bechara, 2005; Cella et al., 2007; Stocco et al., 2009). However, such reason-based decisions were always intentional and explicit.

“*Intuitive*” is therefore a label which appears to capture a decision process reflecting imprecise and emotion-based impressions. We have argued that such EBL systems may pre-empt or guide reason-based choice, when faced with settings involving combinations of a complex problem space; high levels of uncertainty and ambiguity; and laden or infused with affect. Interestingly, this literature potentially links to emotion-based systems of the sort found in psychiatric disorders (Evans et al., 2005), or neurological disorders of emotion regulation (Fotopoulou et al., 2004) – where both groups show impaired understanding in the form of delusional beliefs. These affectively laden biases may perhaps appear without conscious awareness, and lack explicit understanding, even when producing successful outcomes (Damasio, 1994, pp. 187–189; Turnbull et al., 2007).

In sum, one *form* of conscious awareness *does *appear to be readily achieved during the IGT, but this is in the sense of an emotion-based impression: “How much do I like this object?” (Bowman et al., 2005; Evans et al., 2005), though this may also explain why Bechara et al. (1997) report that optimal IGT decision-making operates outside of formal* cognitive *scrutiny.

### PRE-EXISTING AFFECTIVE BIAS ON THE IGT

The IGT is usually regarded as a good simulation of the complexity of real-world decision-making, given that it involves exploratory decisions under both risk *and* ambiguity (e.g., Brand et al., 2007), with shifting contingencies over time. Although other tasks may provide a better psychological dissection of the decision-making processes (e.g., Fellows, 2004; Dunn et al., 2006, 2010; Brand et al., 2007), the IGT is typically regarded as affording an ecologically rich and complex problem space (Damasio et al., 1991; Bechara et al., 1994). Of particular interest is the “balance,” or trade-off, between cognition and affect, as a measure of adaptive task performance (e.g., Manes et al., 2002; Fellows and Farah, 2005; Dunn et al., 2006, 2010; Cassotti et al., 2011). For instance, affective states appear to especially underpin adaptive decisions in the early “opaque” and ambiguous period of the IGT, with the latter phase of the task (as discussed above) more readily informed by conscious awareness of the incentive properties (e.g., Maia and McClelland, 2004; Bowman et al., 2005; Dunn et al., 2006; Brand et al., 2007; Wagar and Dixon, 2007; Stocco and Fum, 2008).

What then of the fact that humans are often biased or predisposed – toward objects, even before they first encounter them? And how does this bias shift over time? Notably, the IGT involves an intrinsic affective *shift,* where initially learned associations require reversal for adaptive behavior on the task (Fellows and Farah, 2005). In many ways, such pre-existing affective biases might be regarded as the psychological foundation of prejudice – for example where humans express a pre-existing negative evaluation, in the absence of knowledge of the object’s intrinsic properties (e.g., Allport, 1954/1979). Overcoming such biases clearly requires reversal of an affectively laden association. Notably, such social biases are understood to be both common and well-established, with the potential to linger outside full awareness (Devine, 1989; Amodio et al., 2003; Gregg et al., 2006). Indeed, the notion that most objects rapidly and automatically evoke affective states is now well-established (e.g., Zajonc, 1980; LeDoux, 1996; Ito and Urland, 2003; Cunningham et al., 2004). Therefore, an ecologically valid *starting* point for the IGT would be a set of objects which are affectively laden, rather than neutral.

A relevant distinction, and one often stressed by the social cognition literature, is that affect can be sourced from an evaluation of the features of the target itself (*integral affect*), or influenced by the background mood state or another unrelated source (*incidental affect*, Pham et al., 2001; Mussweiler and Bodenhausen, 2002; Finucane et al., 2003). Thus, integral affect may result from actual, perceived, or even imaginary characteristics of the decision targets – i.e., with a focus or the object itself. In contrast, incidental affect is sourced from temporary mood states, trait affective states (e.g., anxiety), or transferred from other diffuse sources distinct from the target object (e.g., Cohen et al., 2008).

How might these sources of affect influence complex decision-making? It is likely they are incorporated into an online affective state, which is readily placed to infuse and bias choices (Damasio, 1994; Finucane et al., 2003; Cohen et al., 2008). Here, the literature is patchy in its coverage. The influence of “incidental” affect on judgment and decision-making has been well-studied, suggesting that there are gains in the flexibility and openness of problem-solving in positive mood states (e.g., Isen, 2001), and risk-aversion in states of anxiety (e.g., Raghunathan and Pham, 1999). Indeed, *incidental* affect appears to have important impacts on IGT performance (Schmitt et al., 1999; Carter and Smith-Pasqualini, 2004; Suhr and Tsandis, 2007). However, the primacy (e.g., Zajonc, 1980; LeDoux, 1996) and importance of *integral *(object biased) affect for judgement and decision-making has been less well-investigated (Pham et al., 2001; Finucane et al., 2003). Surprisingly, only a few studies (Hinson et al., 2006; Davies and Turnbull, 2011; Aïte et al., 2013) have assessed integral affective bias in decision-making paradigms like the IGT – although questions of this sort are highly relevant for human social decision-making (e.g., Bechara et al., 1994).

Notably, real-world social behavior involves encountering agents and objects that develop, and ultimately come to *possess*, ambiguous and ambivalent characteristics (e.g., Cacioppo and Berntson, 1994; Cunningham et al., 2003). Thus, an appraisal of a well-known individual (e.g., Tony Blair, Barack Obama, Lance Armstrong, Edward Snowden) may well evoke both negative and positive evaluations, potentially resulting in a net-weighted (heuristic-based) attitude (e.g., Van Harreveld et al., 2004). Ecologically rich paradigms such as the IGT have only recently been employed to examine the impact of affective biases in complex and dynamic decision-making (Hinson et al., 2006; Davies and Turnbull, 2011; Aïte et al., 2013). The following section presents an overview of this research.

## INSIGHTS FROM TASKS INVOLVING AFFECTIVE BIAS

Given the proposed primacy of emotion-based processes (Bechara et al., 1994, 1997), it is perhaps surprising that only three studies have examined affective bias within IGT-style decision-making. While each study uses different variants of the IGT, and a range of affective biases, the data are broadly consistent – demonstrating that pre-existing bias readily impacts complex decision-making (Hinson et al., 2006; Davies and Turnbull, 2011; Aïte et al., 2013).

Using a three-deck variant of the IGT, Hinson et al. (2006) invoked affective bias, by associating task decks with emotional words, which varied according to deck incentives. In the incongruent condition, the “good” deck was labeled with negative words, and “bad” deck labeled with positive words (with the associations reversed for the congruent condition). Additionally, a third “neutral” deck was labeled with emotionally neutral words. As one might predict, incongruent affective bias impaired performance, while congruent bias enhanced decision-making. Thus, the use of stable emotional landmarks from the outset of the task readily biased IGT-style decision-making.

The SCR data collected during the experiments (Hinson et al., 2006) were used to examine the development of discriminating anticipatory SCRs. Incongruent affective bias was found to hinder the development of these physiological markers – with little discrimination in differential SCRs across the three decks. However, in the congruent condition, these anticipatory markers appeared to selectively distinguish between bad deck choices from both good and neutral options. These responses are often viewed as an index of decision biasing “somatic markers” (Bechara et al., 1997). However, in this study the somatic signals produced no causal influence on decision behavior, merely acting as one index of adaptive decision-making (Hinson et al., 2006).

Building on these findings, Davies and Turnbull (2011) investigated features of the classic Gambling Task potentially influenced by affective bias – expanding the topic to include features such as sensitivity to punishment cues (Dalgleish et al., 2004), and the dynamic tracking and weighting of overall deck attitudes (e.g., Van Harreveld et al., 2004; Bowman et al., 2005) that were not explored in the Hinson et al. (2006) experiments. The Davies and Turnbull (2011) tasks introduced affective bias using visual stimuli that were either non-social (International Affective Picture System; Lang et al., 2001) or more socially salient, in the form of racially diverse faces (Tottenham et al., 2009). To control for individual variation, the stimuli were also customized for each participant, by pre-evaluation. As in the Hinson et al. (2006) studies, there was a growing preference for selections from the advantageous decks. Importantly, affective bias altered selection in both congruent and incongruent conditions; especially both experiments demonstrated that affective labels impaired selection behavior specifically under *incongruent* conditions. Additionally, the study (experiment 2) also showed a clear influence of affective bias on *subjective* ratings of task objects over the task.

This sparks the question of *how* such decision-making is changed. Congruency did not influence shifts from the frequently punishing decks, nor did it alter preferences for decks with lower loss frequency. Also, decoupling subjective evaluation data to absolute deck ratings showed that weighting of deck attitudes were unaltered by the congruency manipulation. However, incongruent association *selectively* modulated evaluation of the disadvantageous decks. Indeed, consolidating the importance of awareness of the affective nature of the punishing bad decks, subjective awareness of their incentive nature was strongly associated with adaptive task performance (cf. Maia and McClelland, 2004; Bowman et al., 2005). Such dissociation between deck ratings suggests that deck attitudes in general**were not influenced by affective bias. Instead it appears that sensitivity to *accumulating* losses is a major driving force in IGT decision-making (Christakou et al., 2009; Weller et al., 2007, 2010; cf. Dunn et al., 2010).

## PRE VERSUS POST-DECISIONS

Both of the above studies (i.e., Hinson et al., 2006; Davies and Turnbull, 2011) introduced affective bias at the *decision* level. In contrast, a recent study (Aïte et al., 2013) suggests that placing affective stimuli during the *post-decision* (feedback stage) phase of decision-making also affects performance on IGT. Here, the ability to make an advantageous choice increases when the emotional context is congruent with the feedback, while this is impaired in an incongruent condition. Indeed, facial emotion appears to carry intrinsic incentive value (Shore and Heerey, 2011); therefore presenting bias during the feedback phase should modulate the net decision feedback. For example, providing a reward of $10 with a smile would provide more positive reinforcement than the same reward with a fearful face.

The findings of Aïte et al. (2013) are thus consistent with affective bias influencing the decision process via a range of plausible pathways and mechanisms – both affective and cognitive (e.g., Hinson et al., 2002, 2006; Dunn et al., 2006, 2010). Incongruent affective bias again leads to a robust impact on IGT decision-making (cf. Hinson et al., 2006; Davies and Turnbull, 2011). This would be consistent with the observations made by Davies and Turnbull (2011), and further imply that affective bias within IGT variants disrupts adaptive shifting of decision behavior in the face of changing contingencies (i.e., reversal learning).

A notable inference derived from this study surrounds the use of additional supporting feedback the IGT (and other decision-making paradigms) often present additional feedback with affective value (e.g., smiley faces). Such feedback probably *consolidates* reinforcement of primary incentive feedback, potentially complicating task interpretation (Shore and Heerey, 2011). However, as highlighted by Aïte et al. (2013), the use of such feedback may be unhelpful methodologically, and should therefore be discouraged in IGT experiments.

In sum, a modest number of studies have manipulated the affective loading of IGT tasks – with positive and negative biases, and pre or post-decision influences. All make it clear that affective labeling has a substantial affect on performance, biasing outcome in the direction of the emotion-based influence. Psychophysiological data showed that anticipatory SCRs did not appear to be an important (or necessary) indicator of good decisions. Finally, the awareness of accumulating loss was found to be critical for adaptive task performance (cf. loss aversion; Weller et al., 2007, 2010). In demonstrating these effects, the studies show a useful analogy for the biases of prejudice in everyday decision-making, while demonstrating the flexibility of the IGT as a research tool.

### DISSOCIATING EPISODIC MEMORY AND EMOTION-BASED LEARNING

The remarkably rich literature on the IGT has been a central source of evidence for the role of the frontal lobes in EBL (e.g., Bechara et al., 1994, 1997; Rogers et al., 1999; Bowman and Turnbull, 2004). Indeed, Bechara et al. (1997) original paper especially emphasized the role of ventromedial pre-frontal cortex (VMPFC). A later set of studies narrowed the focus, to investigate which specific frontal regions (right or left, dorsal or lateral, or ventral or medial) played the most significant role in EBL (e.g., Rogers et al., 1999; Duncan and Owen, 2000; see Manes et al., 2002; for a detailed discussion).

However, this focus on the frontal lobes, and thus on executive functions, has potentially ignored the role of other brain areas, and indeed other classes of psychological ability. An especially interesting question is the relationship between EBL and episodic memory. In this section of the paper, we will present evidence from lesion (e.g., Damasio et al., 1996; Bechara et al., 1998; Turnbull and Evans, 2006) and neuroimaging studies (e.g., Patterson et al., 2002; Fukui et al., 2005), to understand the relationship between these key psychological systems.

## EMOTION-BASED LEARNING AND EPISODIC MEMORY

The neurobiology of EBL is far less well understood than that mediating episodic memory. However, an introductory survey of likely brain regions might include a full range of subcortical emotion systems (e.g., Panksepp, 1986, 1998; Davidson and Irwin, 1999; LeDoux, 2000; Rolls, 2000; Calder et al., 2001; Phan et al., 2002; Bechara et al., 2003; Berridge, 2003; Patterson and Schmidt, 2003; Adolphs et al., 2005), as well as the connection between these systems and pre-frontal cortex, in many cases through the VM frontal lobes (Davidson and Irwin, 1999; Bechara et al., 2000a; Bechara, 2004; Anderson et al., 2006).

Consistent with this, studies also suggest that certain emotional-learning processes clearly involve medial prefrontal cortex (e.g., Lane et al., 1997; Reiman et al., 1997). A meta-analysis of neuroimaging studies, for example, suggest that medial prefrontal cortex is involved in emotion-based tasks, while the anterior cingulate and insula are involved when tasks have both emotional and cognitive load (see Phan et al., 2002).

However, lesion-study and imaging findings have suggested that *episodic* memory systems (Tulving, 1972, 1983) particularly include the medial *temporal* lobes and associated structures (e.g., Zola-Morgan et al., 1986; McDonald and White, 1993; Schacter et al., 1995; Nyberg et al., 1996; Schacter et al., 1996; Rugg et al., 1997; Clark and Squire, 1998). In principle, if EBL and episodic memory systems are anatomically independent (Tranel and Damasio, 1993) it should be possible to disrupt one system and leave the other intact.

Evidence of such intact EBL has long been reported, notably in a classic patient with amnesia (Claparède, 1951). In this well-known report, Claparède concealed a pin in his palm, before shaking the hand of an amnesic patient. On the day following this painful episode, the patient refused to shake the physician’s hand, despite having no conscious recollection of the incident (Claparède, 1951; for review see Eichenbaum and Cohen, 2001). Modern and systematic evidence for the claim comes from the work on the profoundly amnesic patient, Boswell (Tranel and Damasio, 1990, 1993; Feinstein et al., 2010). In the experiment (Tranel and Damasio, 1993), Boswell engaged in inter-personal encounters with stooges who played a “good,” “neutral,” or “bad” character in their interactions. After a week, Boswell was shown sets of photographs that included the face of one of the individuals, and an unfamiliar face, and was asked to “Pick the person you would like best?” (p. 83). Naturally, Boswell had no explicit memory of any of the individuals (tested with a free or cued recall). However, when asked to make a forced-choice response, Boswell chose the “good” character almost 80% of the time, and virtually never chose the bad character (Tranel and Damasio, 1993).

What of *complex* learning tasks that also have a reward-based element? Interestingly, some studies have reported relatively normal performance by amnesic patients on the Wisconsin Card Sorting Test (WCST; e.g., Leng and Parkin, 1988; Shoqeirat et al., 1990), and the probabilistic “Weather Prediction” Task (WPT; Knowlton et al., 1994, 1996). A plausible hypothesis is that these tasks also have an emotion-based preference – given that the experimenter provides “correct” or “incorrect” feedback after each trial.

## COMPLEX EMOTION-BASED LEARNING

Empirical evidence from such studies (see also Johnson et al., 1985; Tranel and Damasio, 1989, 1990, 1993) thus suggests that capacity to learn *complex* emotional valence may be retained in profoundly amnesic patients. However, many of reports of the sort described above relate to relatively *simple* patterns of emotional valence learning (uniformly good versus uniformly bad, e.g., Tranel and Damasio, 1993; Feinstein et al., 2010), rather than the more sophisticated patterns of valence which characterize everyday life (e.g., Barraclough et al., 2004). As noted earlier, it is precisely this complicated pattern of reward and punishment that the IGT was designed to assess (Bechara et al., 2000a).

In this context, Turnbull and Evans (2006) measured the IGT performance of a profoundly amnesic patient (SL) who had suffered a posterior cerebral artery stroke, producing profound amnesia. On the IGT, SL performed at a comparable level to controls, across a 3-week period, where each week his performance was no different to (or in one case much better), than controls. This learning was also seen despite the fact that the reward-contingency pattern was shifted between sessions (c.f. Fellows and Farah, 2003), and that SL was unable to explicitly recall any aspect of the previous sessions, or recognize the examiner – evidence suggesting that EBL was preserved.

Thus, complex EBL can remain intact despite profound amnesia – though this effect is not universal. Turnbull and Evans (2006) patient may have been a relatively rare example of such a powerful dissociation. Gutbrod et al. (2006) report patients with lesions to the basal forebrain (*N* = 5) or medial temporal lobe (*N* = 6) who performed the IGT. Here two patients *did* develop a behavioral preference, though the other nine patients performance remained at chance. In a further study, Gupta et al. (2009) investigated five patients who had bilateral hippocampal damage, and reported that no patients developed a preference for advantageous over disadvantageous choice.

Further evidence of preserved implicit EBL has been reported in patients with Alzheimer’s disease (AD), another pathology targeting the medial temporal lobe (e.g., Winograd et al., 1999; Blessing et al., 2006). For example, Evans-Roberts and Turnbull (2011) investigated EBL using the IGT in a patient with dementia of the Alzheimer’s type – who had profound impairment of both verbal and visual recent episodic memory, and completed the Gambling task over three weeks (as in Turnbull and Evans, 2006). Mr. A again performed consistently above chance, an effect which seems unlikely to be a result if the more “liberal” response bias of Alzheimer’s patients (Budson et al., 2006).

An interesting related finding was the remarkably good performance, in SL’s recognition of paired-associate items (Turnbull and Evans, 2006). He had comprehensively failed to bring even a single one of these pairs to conscious recall on any his 40 previous exposures to the pairs, but nevertheless appeared to have encoded at least some aspect of a memorial linkage between them. One explanation might be that he had stored some emotional marker associated with each pair (“rose–bag,” good; “elephant–glass,” bad). Another possibility might be that the previously tested items had acquired some positive emotional valence through the “mere-exposure” effect (Zajonc, 1980; see Turnbull and Evans, 2006 for detailed report of SL).

These data support the growing evidence that there are multiple memory systems in the brain, especially supporting an anatomical and functional dissociation between episodic (e.g., Schacter and Tulving, 1994; Schacter et al., 2000) and emotion-based memory (Tranel and Damasio, 1993; Damasio, 1994; Panksepp, 1998; see Eichenbaum and Cohen, 2001; Phan et al., 2002 for reviews). These findings are consistent with performance of amnestic patients in other non-declarative memory and learning system (e.g., motor learning). The evidence clearly suggests that EBL systems appear to encode more sophisticated patterns of valence learning than have previously been reported, and sustain these over substantial periods of time, especially in patients with “hippocampal” amnesia (Turnbull and Evans, 2006).

## CLINICAL IMPLICATIONS

These findings have a range of potentially important clinical implications. For example the Evans-Roberts and Turnbull (2011) study on preserved EBL in dementia clearly supports claims from the “person-centered” literature (e.g., Kitwood, 1997; Sabat and Collins, 1999) – that in spite of progressive memory loss (Blessing et al., 2006) patients with AD are able to learn and retain emotion-based knowledge. Unfortunately, the behavior of many of those who care for patients with AD is less than optimal (Sabat and Collins, 1999). Such carers may hold the opinion that they can perhaps speak critically of such patients, because they will inevitably forget the experience. The systematic findings reported above suggest that patients with AD may retain emotion-based memories, which may have direct impact on interpersonal relationships with patients with memory loss, both in a personal and therapeutic context.

In addition, the finding of preserved emotional learning in the face of profound amnesia is of some interest in the context of infantile amnesia. It is well established that humans have poor, or non-existent, episodic memory for the first few years of life (Freud, 1905; Dudycha and Dudycha, 1941; Sheingold and Tenney, 1982 for review see Pillemer and White, 1989). Indeed, there is some consensus that the earliest adult autobiographical memories are for events that occurred between 2.5 and 4 years of age (Waldfogel, 1948; Wetzler and Sweeney, 1986; Bruce et al., 2000; MacDonald et al., 2000).

Some researchers posit that language development plays a crucial causal role in such childhood amnesia (Allport, 1937; Schachtel, 1947; Simcock and Hayne, 2002; see also Hayne, 2004 for a review). While, modern neuroscientific accounts of the phenomenon stress especially the late development of hippocampal (conscious) memory systems (for further discussion, see Yovell, 2000; see also Jacobs and Nadel, 1985; Turnbull and Evans, 2006). However, surely these children are learning from this period of early childhood? It is now clear that infants *do* process a well-developed capacity for learning of *emotional* valence in relation to objects, for example, the quality of attachment relationships with specific adults (Winnicott, 1960; Bowlby, 1969; Ainsworth et al., 1978; Ainsworth, 1985; Fonagy et al., 1991a,b). The empirical evidence from childhood amnesia studies suggests EBL systems might be available to infants possibly much before the hippocampus-based systems develop.

Interestingly, this issue may also be important for our understanding of the mechanisms of psychotherapy. It has been suggested that aspects of the therapeutic alliance might (for example) be mediated by emotion-based non-episodic memory systems (Turnbull et al., 2006). In principle, this topic could be investigated through the study of neurological patients with amnesia in a psychotherapeutic setting (Turnbull et al., 2006). In a report of a patient with severe and stable amnesia, Mr. N (see Kaplan, 1994, pp. 590–624 for details), there is at least some evidence that the patient shows therapeutic gains from the interaction with the therapist (Turnbull et al., 2006). Moore et al. (2012) report a similar finding. These preliminary data suggest that during psychotherapy the interpersonal properties of the therapeutic relationship may still exist in patients with profound amnesia, suggesting that the therapeutic alliance may be mediated by a class of memory system that is separate to that of episodic recall.

## CONCLUSION

Summarizing the literature over the last two decades, it is evident that EBL, in the face of a complex ambiguous decision-making landscape, is an important psychological process that occurs rapidly, and is remarkably flexible. This specific form of learning contributes to the scientific understanding of psychological phenomena such as intuition, prejudice that were long ignored, and often difficult to define functionally.

For much of its history, psychological science focused on rational choice, rather that the less well-specified and emotion-based intuitive aspects of human choice (Gilhooly and Murphy, 2005). These later systems are clearly enormously important for human beings, and this paper has reviewed our growing understanding of range of important issues: the flexibility of theses systems, their access to conscious awareness, their relationship to episodic memory, their role in prejudice, and a number of potentially important implications for psychotherapy and care of the elderly. However, this strand of research is clearly still only in the early stages of development, and we anticipate a range of future discoveries on this scientifically important topic.

## Conflict of Interest Statement

The authors declare that the research was conducted in the absence of any commercial or financial relationships that could be construed as a potential conflict of interest.
